# The anti-myeloma activity of a novel purine scaffold HSP90 inhibitor PU-H71 is via inhibition of both HSP90A and HSP90B1

**DOI:** 10.1186/1756-8722-3-40

**Published:** 2010-10-26

**Authors:** Saad Z Usmani, Robert D Bona, Gabriela Chiosis, Zihai Li

**Affiliations:** 1 Myeloma Institute for Research & Therapy, University of Arkansas for Medical Sciences, Little Rock, AR, USA; 2 Carole and Ray Neag Comprehensive Cancer Center, Lea's Center for Hematologic Disorders, University of Connecticut Health Center, Farmington, CT, USA; 3 Department of Molecular Pharmacology & Chemistry, Memorial Sloan Kettering Cancer Center, New York, NY, USA; 4 Department of Immunobiology & Cancer Immunology, Hollings Cancer Center, Medical University of South Carolina, Charleston, SC, USA

## Abstract

**Background:**

Heat shock protein 90 (HSP90) inhibitors have emerged as a promising class of anti-cancer drugs in both solid and hematologic malignancies. The HSP90 family includes the cytosolic HSP90 (HSP90AA1), the ER paralogue gp96 (HSP90B1) and the mitochondrial member TRAP1 (HSP90L). We evaluated the *in vitro *anti-tumor activity and mechanism of action of PU-H71, a novel purine scaffold HSP90 inhibitor in human multiple myeloma cell lines.

**Methods:**

Multiple human myeloma cell lines including cells that are resistant to corticosteroids and bortezimab were treated with PU-H71, followed by analysis of cell viability, cell cycle progression and apoptosis, by flow cytometry and caspase 3 immunoblot. Induction of unfolded protein response was studied by XBP-1 s immunoblot. The role of gp96 was further assessed by small hairpin RNA knockdown of gp96 before treatment with PU-H71.

**Results:**

PU-H71 has potent *in vitro *anti-myeloma activity in both drug-sensitive and drug-resistant cell lines. PU-H71 activates the unfolded protein response and induces caspase-dependent apoptosis. The stable gp96 knockdown human myeloma cell line was found to be more resistant to PU-H71 and other HSP90 inhibitors including 17-AAG and 17-DMAG, even though these cells are more sensitive to conventional anti-myeloma drugs.

**Conclusion:**

We conclude that PU-H71 is a promising drug for the treatment of myeloma. Our finding further suggests that PU-H71 and the geldanamycin analogues work in part by inhibiting the endoplasmic reticulum gp96 along with the cytosolic HSP90.

## Introduction

Multiple myeloma is a clonal plasma cell malignancy accompanied by characteristic bone lesions, cytopenias, renal insufficiency and immune deficiency. The last decade has witnessed significant advances in anti-myeloma therapy with median survival extending from 2-3 years to over 7 years for patients younger than 50 years [1].

Heat shock protein 90 (HSP90) inhibitors are an emerging class of targeted agents in cancer therapy. HSP90 inhibition would make intuitive sense as anti-tumor therapy in cell types that depend on sustained protein homeostasis for their survival. A number of HSP90 inhibitors have demonstrated anti-myeloma activity in pre-clinical studies and at least three compounds have been evaluated in Phase I trials for relapsed/refractory myeloma [2-5]. PU-H71 is a novel purine scaffold HSP90 inhibitor that has shown pre-clinical activity in triple negative breast cancer [6], Bcl6 dependent lymphoma [7], hepatocellular carcinoma [8] and myeloproliferative disorders [9].

HSP90 family of proteins are ubiquitous molecular chaperones that are involved in folding, activation, maturation and assembly of many proteins (referred to as HSP90 client proteins or HSP90 clientele) that include essential mediators of signal transduction and cell cycle progression [10]. The mammalian HSP90 family members include the cytosolic HSP90, the HSP90 paralogue gp96 (also known as grp94, endoplasmin, HSP90B1) in the endoplasmic reticulum (ER) and the mitochondrial protein TRAP1. There have been recent significant progresses in the understanding of both the structure and function of gp96. It serves as an obligate master chaperone for multiple Toll-like receptors [11,12] and integrins [13,14], neither of which could function properly in the absence of gp96. More recently, gp96 has been observed to play a critical role in lymphopoeisis in that deletion of gp96 leads to a transitional block from pro-B to pre-B cells and the inability of thymocytes to develop beyond the CD4(-)CD8(-) stage [14]. gp96 also maintains the fidelity of the endoplasmic reticulum protein synthesis by mediating the unfolded protein response (UPR) [15]. It shares ~50% homology at the amino acid level with its cytosolic HSP90 paralogue, with a similar domain organization consisting of an N-terminal ATP-binding domain, a charged middle domain and a C-terminal homodimerization domain [16].

The unfolded protein response (UPR) is a highly conserved eukaryotic protein homeostasis mechanism that is especially important for secretory cell types (e.g., hepatocytes, plasma cells, etc.) [17]. In response to cellular stress, UPR leads to increased ER chaperones such as grp78, gp96 and calreticulin to deal with the increased load of unfolded and nascent proteins in the ER. In response to sustained cellular stress, the UPR activates the apoptotic pathway. It has been previously demonstrated that, a geldanamycin derived HSP90 inhibitor, can activate the unfolded protein response in myeloma cells [18].

Herein, we evaluated the *in vitro *anti-myeloma activity of PU-H71, a novel purine scaffold HSP90 inhibitor. We also determined if the anti-tumor activity of HSP90 inhibitors is achieved via targeting both cytosolic HSP90 and the endoplasmic reticulum HSP90 paralogue gp96.

## Materials and methods

### Cell lines

We studied a panel of previously established human MM cell lines and sublines (MM-1 S, MM-1R, RPMI-8226/S, RPMI-8226/Dox40, INA-6, NCI-H929, OPM-1 U266). The dexamethasone (Dex)-sensitive parental line MM-1 S and its Dex-resistant subline MM-1R cells were kindly provided by Dr. Steven Rosen (Northwestern University, Chicago, IL); the chemo-resistant subline RPMI-8226/Dox40 (doxorubicin-resistant) cells were provided by Dr. William Dalton (Lee Moffitt Cancer Center, Tampa, FL); OPM-1cells were provided by Dr. Brad Thompson; INA-6 cells were provided by Renate Burger (University of Erlangen-Nuernberg, Germany); RPMI-8226/S, NCI-H929 and U266 cells were purchased from American Type Cell Culture (ATCC). All cells were cultured in RPMI 1640 medium (Life Technologies, Grand Island, NY) supplemented with 10% fetal bovine serum, L-glutamine, penicillin, and streptomycin (Life Technologies), except for INA-6 and XG-1 cells, which were cultured in media supplemented with 20% fetal bovine serum (FBS) and 2.5 ng/mL of human recombinant IL-6.

### Ex vivo drug sensitivity assays

Cell survival of PU-H71 treated cells (1 × 10^5 ^cells plated in triplicate) was examined using trypan blue staining followed by cell count for viability using a hemocytometer.

### HSP90B1 (gp96) knockdown by short hairpin RNA (shRNA)

Empty vector (EV) and gp96 pLKO shRNA vectors were obtained from Open Biosystems (Huntsville, AL). Recombinant lentivirus was produced by transient transfection of 293T cells following a standard protocol [12]. After 48 hours, myeloma cells were incubated with culture supernatants from 293T cells containing crude virus. Spin infection was achieved by centrifugation at 1900 g for 1.5 h at 32°C to facilitate viral transduction. Cells were then fed with fresh media and allowed to recover for 48-72 hours before being subjected to puromycin selection.

### Reagents

Most antibodies (Abs) used for flow cytometry were obtained from Biolegend (Mountain View, CA), eBioscience (San Diego, CA), except Abs against p-Akt, p-IKB, and p-eIF2α (Cell Signaling Technology, Danvers, MA) and HSP90b1 (Stressgen, Victoria, BC). The active geldanamycin analog 17-allylamino-17-demethoxy-geldanamcyin (17-AAG, NSC 330507) was obtained from the Drug Synthesis and Chemistry Branch, Developmental Therapeutics Program, National Cancer Institute (Bethesda, MD); Bortezomib was purchased from LC Laboratories (Woburn, MA). All other chemicals were obtained from Sigma-Aldrich (St Louis, MO).

### Flow cytometry

Ab staining, flow cytometry instrumentation, and data analysis were performed essentially as described without significant modifications [13]. In brief, single-cell suspensions were made, washed, and blocked with staining buffer containing serum. Cells were then incubated with primary Ab, followed by incubation with fluorochrome-labeled secondary Ab. Immediately before instrumentation, propidium iodide (PI) was added to stain and exclude dead cells. Cells were finally acquired with a FACSCalibur (BD Biosciences). For intracellular staining, cells were fixed and permeabilized using ice-cold 70% EtOH. Nonspecific binding was blocked with 10% normal goat serum. Fluorescence-activated cell sorter data were analyzed on FlowJo software (TreeStar). For DNA content by PI staining, cells were fixed and permeabilized with 70% ice cold ethanol followed by PBS wash. Cells were then incubated for 30 minutes with 50 ml of RNAase A (1 mg/ml stock, Sigma) and 50 ml of PI solution (1 mg/ml, Sigma) before being analyzed on FACSCalibur (BD Biosciences).

### Immunoblot

Myeloma lysates were prepared with radioimmunoprecipitation assay (RIPA) lysis buffer (0.01 M sodium phosphate (pH 7.2), 150 mM NaCl, 2 mM EDTA, 1% Nonidet P-40, 1% sodium deoxycholate, and 0.1% SDS) and quantitated by Bradford protein assay (Bio-Rad). 25-50 micrograms of total lysate were denatured by boiling for 5 min in the presence of 0.1 M DTT and SDS-loading buffer, followed by resolution on a 10% SDS-PAGE and transfer to a polyvinylidene difluoride membrane. After blocking, membranes were blotted with appropriate primary and horseradish peroxidase-conjugated secondary Abs and developed with chemiluminescent substrate (Pierce Chemical). β-actin was blotted (AC-74; Sigma-Aldrich) as a loading control.

### Statistical analysis

Error bars represent the standard error of the arithmetic mean (SEM). Student *t *test and ANOVA were used for statistical analysis. Values of *P *less than 0.05 were considered to represent statistically significant differences.

## Results

### PU-H71 has potent *in vitro *anti-myeloma activity

**Figure 1:**
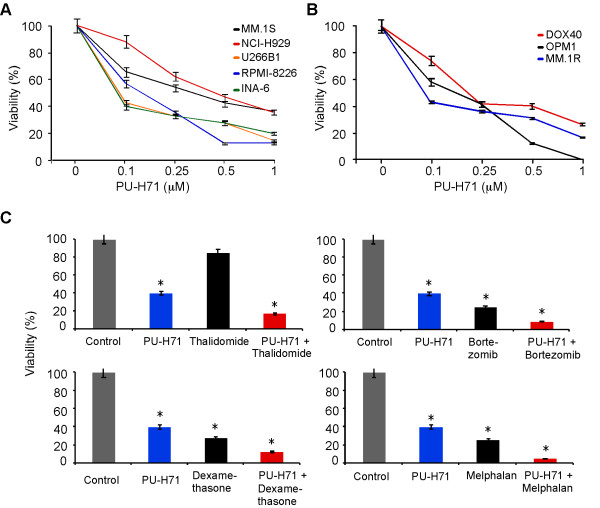
**In vitro anti-myeloma activity of PU-H71**. **(A) **Drug-sensitive human myeloma cell lines were treated with PU-H71 for 24 hours followed by cell viability analysis. **(B) **Same as (A) except that drug-resistant cell lines are used. **(C) **Synergistic effects of PU-H71 (100 nM) with melphalan (1 μM), dexamethasone (1 μM), thalidomide (1 μM) and bortezomib (100 nM) on INA-6 cells treated for 24 hours. * *p < 0.05*

To determine the anti-myeloma activity of this new class of HSP90 inhibitor, we exposed a panel of human myeloma cell lines to increasing molar concentrations of PU-H71 (Figures 1. We found that PU-H71 has potent anti-tumor effects in both drug-sensitive (MM-1 S, RPMI-8226/S, INA-6, NCI-H929 and U266) and drug-resistant (MM-1R, RPMI-8226/Dox40, OPM-1) human myeloma cell lines (HMCLs). The observed myeloma cell death was dose-dependent and reproducible. The half maximal concentration of PU-H71 to exert anti-myeloma killing effect (IC50) was in the range of 100 nM to 300 nM at 24 hours. These IC50 values are comparable to the published data in breast cancer [6] and Jak2/MPL mutant cells (~100 nM) [9], but significantly less than Bcl-6-dependent lymphoma cells ( > 1 μM) [7]. Importantly, PU-H71 also demonstrated pronounced *in vitro *synergy with other anti-myeloma drugs including dexamethasone, melphalan, thalidomide and bortezomib (Figure 1C).

### PU-H71 causes cell cycle arrest in the G1-S phase

**Figure 2:**
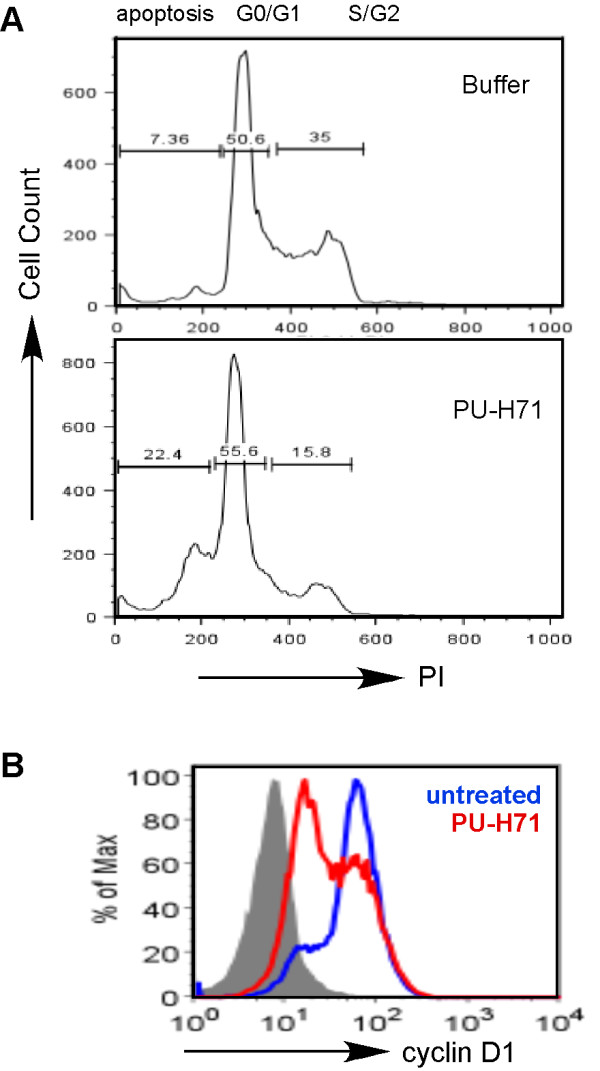
**Effect of PU-H71 on cell cycle**. **(A) **DNA content analysis by flow cytometry performed on MM.1R and RPMI-8226/DOX40 cells exposed to PU-H71 (100 nM) for 24 hours. **(B) **Intracellular staining for cyclin D1 in MM.1R cells after 24-hour treatment with PU-H71 (100 nM).

We studied the effect of PU-H71 on cell cycle progression in the human myeloma cell lines at 24 hours of drug exposure. We found that PU-H71 treatment led to a decline in the proportions of cells in the G2-M phase demonstrated by flow cytometric analysis of DNA content (Figure 2A). This was accompanied by the reduction in the cyclin D1 expression at the protein level (Figure 2B), measured by flow cytometry after intracellular stain. These results suggest strongly that PU-H71 inhibits cell cycle progression.

### PU-H71 activates the unfolded protein response and induces caspase dependent apoptosis

**Figure 3:**
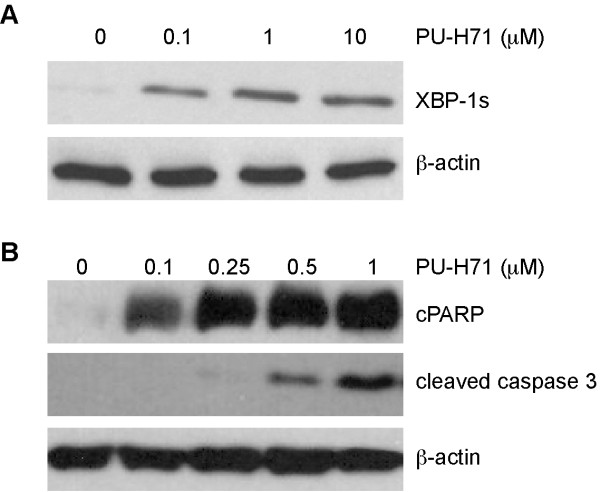
**Effect of PU-H71 on the unfolded protein response (UPR) and apoptosis**. **(A) **Immunoblot of XBP1 s on 24 hour treated RPMI-8226/DOX40 cells. **(B) **Immunoblot on 24-hour treated MM.1R cell lysate for cleaved caspase 3 and cleaved PARP (cPARP).

We exposed the dexamethasone-resistant cell line MM.1R to increasing concentrations of PU-H71 to evaluate the unfolded protein response and the mechanism of apoptosis. PU-H71 upregulates spliced XBP1 consistent with activation of the unfolded protein response [17] (Figure 3A). It was observed that PU-H71 treatment resulted in accumulation of the cleaved caspase 3 in a dose-dependent manner (Figure 3B). This was accompanied by increase in cleavage of poly(ADP-ribose) polymerase (PARP), as a downstream consequence of activated caspase-3 [19]. In combination, these observations suggest that PU-H71 can cause myeloma cell death by activation of the unfolded protein response and the caspase-dependent apoptotic pathway.

### PU-H71 has decreased anti-myeloma activity in the gp96 knockdown human myeloma cells

**Figure 4:**
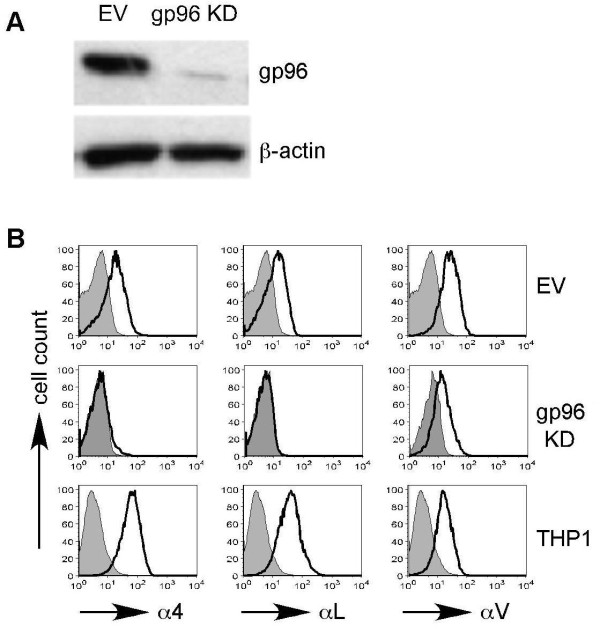
**Generation of gp96 knockdown RPMI-8226 cell line**. **(A) **Immunoblot analysis of gp96 after stable transduction with empty vector (EV) or gp96 shRNA lentiviral vector (gp96 KD). **(B) **Loss of surface integrins expression (open histogram) by flow cytometry in gp96 KD cells. Shaded histograms represent background staining with isotype-control antibody. THP-1 cells were used for positive control for this experiment.

**Figure 5:**
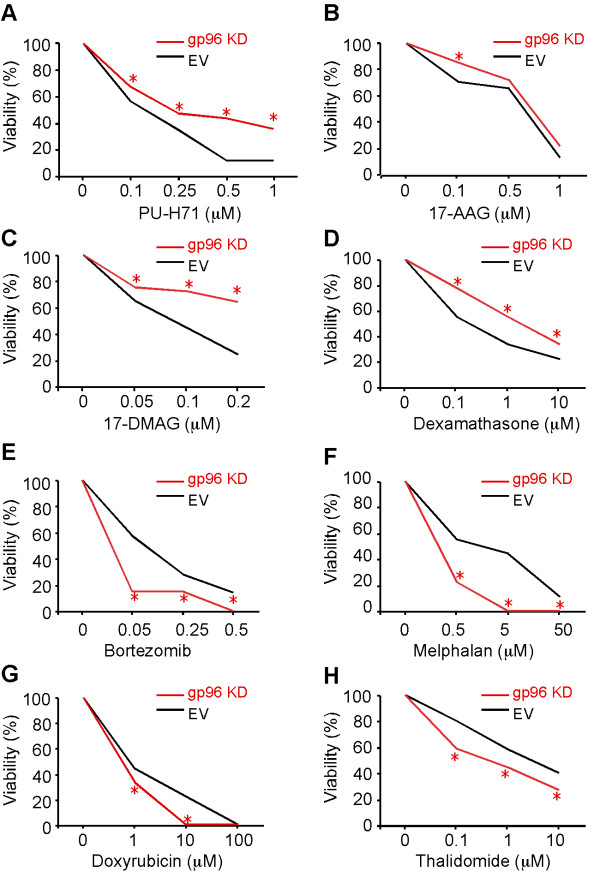
**Anti-myeloma effects of drugs against gp96 knockdown cell line**. **(A) **PU-H71. **(B) **17-AAG. **(C) **17-DMAG. **(D) **Dexamethasone. **(E) **Bortezomib. **(F) **Melphalan. **(G) **Doxorubicin. **(H) **Thalidomide. All drug treatment was for 24 hours. * *p < 0.05*.

To determine if PU-H71 exerts its anti-myeloma activity is dependent on targeting gp96, we stably knocked down gp96 from RPMI-8226 using a lentiviral vector-based shRNA vector (Figure 4A). Consistent with the roles of gp96 in chaperoning integrins, the knockdown cells expressed significantly less surface integrins including α4, αL and αV integrins, comparing with cells treated with empty vector (Figure 4B). Upon exposing these cells to increasing concentrations of PU-H71, the gp96 knockdown cells demonstrated increased resistance to PU-H71, as well as to two other pan-HSP90 inhibitors: geldanamycin analogues 17-AAG and 17-DMAG (Figure 5A-C). Interestingly, the sensitivity to dexamathasone-mediated anti-myeloma effect was also reduced with gp96 knockdown (Figure 5D). To rule out that gp96 knockdown caused general drug resistance, we also treated cells with increasing doses of melphalan, doxorubicin, bortezomib and thalidomide (Figure 5. We found that the gp96 knockdown cells were more susceptible than control cells to these agents. Taken together, these observations suggest that PU-H71 and the geldanamycin analogues exert their anti-tumor activity via targeting both cytosolic HSP90 and gp96.

## Discussion

The cytosolic HSP90 has a diverse list of client proteins that participate in pathways linked to all six hallmarks of cancer development proposed by Weinberg [5,20]. A number of ATP-competitive inhibitors, including the geldanamycin derived 17-AAG, 17-DMAG and IPI-504, have been developed and investigated for the treatment of both solid and hematologic malignancies. The geldanamycin derivatives have demonstrated significant dose limiting non-hematopoietic toxicities, limiting their clinical efficacy [5]. This has led to development of purine scaffold, non-benzoquinone HSP90 inhibitors [21]. Preclinical studies with PU-H71 in triple-negative breast cancer [6] and diffuse large B-cell lymphoma [7] have demonstrated considerable efficacy with limited hematopoietic or non-hematopoietic toxicity. However, the efficacy of PU-H71 has not been investigated in multiple myeloma. We exposed a panel of human multiple myeloma cell lines to escalating doses of PU-H71 and discovered it to be highly active *in vitro*. The anti-tumor activity was observed in both drug-sensitive and drug-resistant cell lines. PU-H71 showed comparable anti-myeloma activity in IL-6 dependent cell line INA-6 and the other IL-6 independent cell lines. PU-H71 also demonstrated *in vitro *synergy with other conventional anti-myeloma drugs. Mechanistically, we found that PU-H71 led to activation of the UPR and apoptosis via the caspase-dependent pathway.

The past decade brought forth valuable insights on the endoplasmic reticulum HSP90 family member, i.e., gp96 (HSP90B1, grp94). On the one hand, because of the nature of gp96 to chaperone antigenic peptides to antigen-cross-presentation pathway [22], vaccinations with purified tumor-derived gp96-peptide complex have demonstrated promising pre-clinical and clinical results in a number of malignancies, including multiple myeloma [23]. On the other hand, endogenous gp96 is the master chaperone for most integrins (including α4, αV and αL) [14] and is one of the key mediators in the unfolded protein response. gp96 blockade, therefore, can (a) disrupt the integrin-dependent cell adhesion mediated drug resistance in multiple myeloma; (b) upset protein homeostasis and compromise the efficiency of the unfolded protein response. In this study, we established a stable gp96 knockdown human myeloma cell line to evaluate the *in vitro *activity of anti-myeloma drugs in the absence of gp96. We found, as predicted, that gp96 knockdown cells were more sensitive to bortezomib which further compromises protein homeostasis. gp96 knockdown cells were also more susceptive to killing mediated by genotoxic drug melphalan and doxorubicin, and to thalidomide that has a yet not fully understood mechanism of action. The anti-myeloma activity of PU-H71 and the geldanamycin analogues (17-AAG, 17-DMAG), which are all pan-HSP90 inhibitors, was, however, significantly reduced in the absence of gp96. This data suggests that HSP90 inhibitors exert their acute cytotoxic activity via inhibiting not only cytosolic HSP90, but also gp96. The interaction between HSP90 inhibitors and gp96 might trigger apoptosis that is dependent on the unfolded protein response pathway. In the absence or low level of gp96 in the chronic setting (as in the case of shRNA knockdown), an unknown compensatory mechanism might develop to overcome the defective protein quality control associated with gp96 loss. Such a mechanism might render cells less dependent on even the cytosolic HSP90. Interestingly, dexamethasone also had reduced efficacy in the absence of gp96, suggesting that TLR-dependent signaling might participate in the glucocorticoid-mediated apoptosis. Further study is necessary to clarify this point.

## Conclusions

In summary, our study highlights the *in vitro *anti-myeloma activity of PU-H71, a novel purine scaffold HSP90 inhibitor. We also demonstrated that the anti-myeloma effect of HSP90 inhibitors including PU-H71 is via targeting both cytosolic HSP90 and the ER HSP gp96. A phase I study to test the clinical utility of PU-H71 in patients with refractory myeloma is warranted.

## Competing interests

The authors declare that they have no competing interests.

## Authors' contributions

SZU and ZL conceived, designed and implemented the study, and drafted the manuscript.

RB and GC participated in the implementation of the study, and the acquisition, analysis and interpretation of data.

All authors have read and approved the final manuscript.
